# A novel galactolipase from a green microalga *Chlorella kessleri*: purification, characterization, molecular cloning, and heterologous expression

**DOI:** 10.1007/s00253-017-8713-7

**Published:** 2018-01-03

**Authors:** Shuhei Hashiro, Koyu Fujiuchi, Daisuke Sugimori, Hisashi Yasueda

**Affiliations:** 1 Institute for Innovation, Ajinomoto Co., Inc., 1-1 Suzuki-cho, Kawasaki-ku, Kawasaki, 210-8681 Japan; 2 Department of Symbiotic Systems Science and Technology, Graduate School of Symbiotic Systems Science and Technology, Fukushima University, 1 Kanayagawa, Fukushima, 960-1296 Japan

**Keywords:** *Chlorella kessleri*, Galactolipase, Characterization, Genomic structure, cDNA cloning, Heterologous expression

## Abstract

We have identified an enzyme, galactolipase (ckGL), which hydrolyzes the acyl ester bond of galactolipids such as digalactosyldiacylglycerol (DGDG), in the microalga *Chlorella kessleri*. Following purification of the enzyme to electrophoretic homogeneity from cell-free extract, the maximum activity toward DGDG was observed at pH 6.5 and 37 °C. ckGL was Ca^2+^-dependent enzyme and displayed an apparent molecular mass of approx. 53 kDa on SDS-PAGE. The substrate specificity was in the order: DGDG (100%) > monogalactosyldiacylglycerol ≈ phosphatidylglycerol (~ 40%) > sulfoquinovosyldiacylglycerol (~ 20%); the enzyme exhibited almost no activity toward glycerides and other phospholipids. Gas chromatography analysis demonstrated that ckGL preferably hydrolyzed the *sn*-1 acyl ester bond in the substrates. The genomic DNA sequence (5.6 kb) containing the ckGL gene (designated *glp1*) was determined and the cDNA was cloned. *glp1* was composed of 10 introns and 11 exons, and the 1608-bp full-length cDNA encoded a mature ckGL containing 475 amino acids (aa), with a presequence (60 aa) containing a potential chloroplast transit peptide. Recombinant functional ckGL was produced in *Escherichia coli*. Although the deduced aa sequence of ckGL contained the typical GXSXG motif of serine hydrolases together with conserved histidine and aspartate residues which would form part of the catalytic triad of α/β-hydrolases, ckGL showed no significant overall similarity with known lipases including GLs from *Chlamydomonas reinhardtii* and *Aspergillus japonicus*, indicating that ckGL is a novel GL. ckGL, with high specificity for DGDG, could be applicable to food processing as an enzyme capable of improving material textures.

**Electronic supplementary material:**

The online version of this article (10.1007/s00253-017-8713-7) contains supplementary material, which is available to authorized users.

## Introduction

**Fig. 1:**
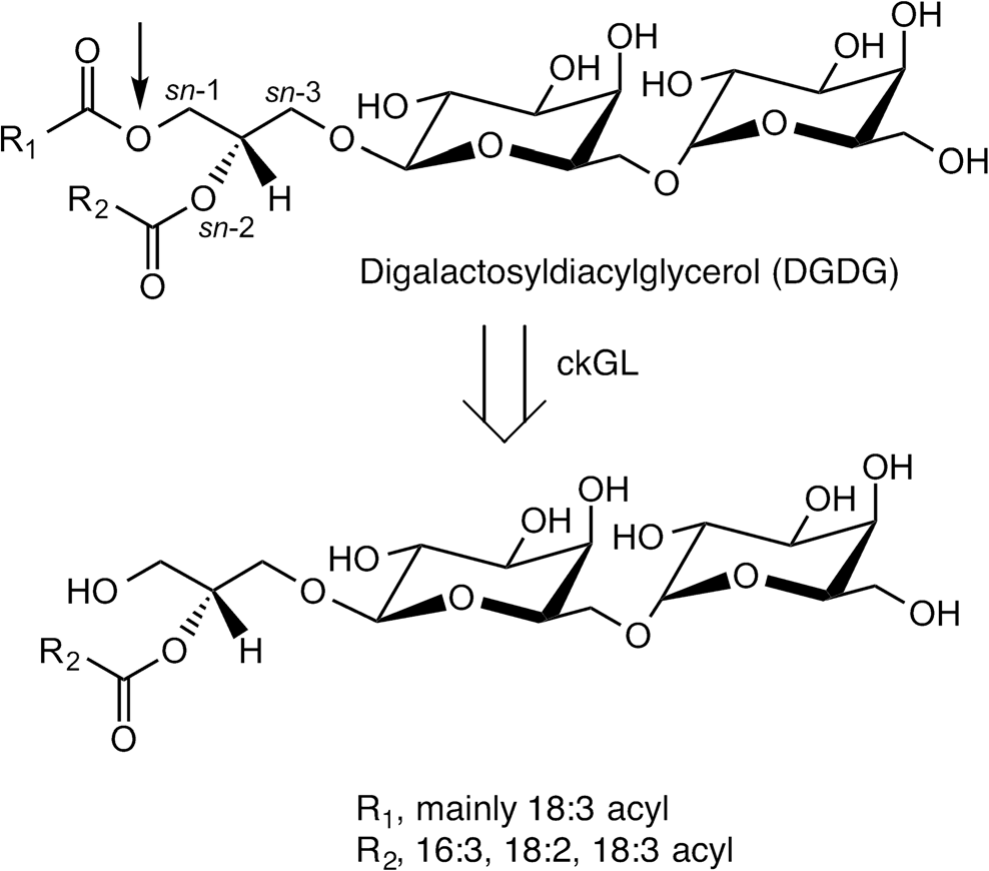
Hydrolysis of digalactosyldiacylglycerol (DGDG) by ckGL. ckGL preferentially catalyzes hydrolysis of DGDG at the *sn*-1 position (indicated with a thin arrow)

Galactolipase (GL) [EC 3.1.1.26] is an enzyme that catalyzes hydrolytic cleavage of acyl ester bonds of galactolipids such as monogalactosyldiacylglycerol (MGDG) and digalactosyldiacylglycerol (DGDG), forming free fatty acids (FFAs), digalactosylglycerol, or lysogalactolipids (LysoGL). LysoGLs include monogalactosyl-1- or -2-acylglycerol (1- or 2-MGMG) and digalactosyl-1- or -2-acylglycerol (1- or -2-DGMG) (Fig. 1). In green plants, galactolipids are principal components of chloroplast and thylakoid membranes. Recently, GL and its reaction products LysoGL and mono- and di-galactosylglycerol have become of interest in the food, cosmetic, and pharmaceutical industries, as well as the chemical industry, because of their useful biochemical properties and functions. For example, it has been reported that an enzyme capable of hydrolyzing a glycolipid improves the handling ability of flour dough and the textures of bread and other baked products (Bojsen et al. 2005), and also that LysoGL can be used as a natural emulsifier for quality improvement of wheat flour (Tsukazaki et al. 2012).

Since GL from the leaves of *Phaseolus multiflous* was first identified as a lipase catalyzing the hydrolysis of MGDG and DGDG (Sastry and Kates 1964), GLs have been found in many plants, mammals, fungi, microalgae, and bacteria (http://brenda-enzymes.info/enzyme.php?ecno=3.1.1.26). Recently, Li et al. reported a GL (PGD1) from *Chlamydomonas reinhardtii* (Li et al. 2012), and a galactolipid- and phosphatidylcholine-hydrolyzing lipase from *Aspergillus japonicus* was also described (Tsukazaki et al. 2012). However, there is only limited information on the structure and characterization of GLs, and their detailed functions remain to be fully elucidated.

*Chlorella kessleri* is a green, coccoid alga that has a rapid growth rate and potential for mass production of starch and lipids (Mizuno et al. 2013; Li et al. 2013). These properties make this microalga an attractive candidate for algal biofuel production. In the process of lipid extraction from cultured algal bodies of *C. kessleri*, we previously found a relatively high GL activity in the cell extracts (Hashiro et al. 2014), and, therefore, we started to study the GL from *C. kessleri* (ckGL).

Here, we report the purification and characterization of ckGL, and also the molecular cloning of the ckGL gene (named *glp1*), as well as its efficient heterologous production in *Escherichia coli*. To the best of our knowledge, no previous information is available concerning a GL having highly specific activity toward DGDG. Therefore, the identification and characterization of ckGL helps to describe a new pathway for galactolipid metabolism, in particular in microalgae and plants. Moreover, the characterization of GL certainly demonstrates differences between GLs and lipases with regard to function, substrate recognition mechanism, and biochemical roles. We also expect our investigation to contribute to fundamental study in food-material processing technology via the application of GL.

## Materials and methods

### Materials

Salt mixture of Gamborg’s B-5 medium (B5 medium) was purchased from Wako Pure Chemical Industries, Ltd. (Osaka, Japan). 1,2-Diacyl-3-β-D-galactosyl-*sn*-glycerol (MGDG), 1,2-diacyl-3-O-(α-D-galactosyl1-6)-β-D-galactosyl-*sn*-glycerol (DGDG), and sulfoquinovosyl diglyceride (SQDG) were from Larodan Fine Chemicals AB (Solna, Sweden). 1,2-Dimyristoyl-*sn* -glycero-3-phosphate, monosodium salt (DMPA), 1-palmitoyl-2-oleoyl-*sn* -glycero-3-phosphocholine (POPC), 1-palmitoyl-2-oleoyl-*sn*-glycero-3-phosphoethanolamine (POPE), 1-palmitoyl-2-oleoyl-*sn*-glycero-3-phospho-L-serine (POPS), and 1-palmitoyl-2-oleoyl-*sn*-glycero-3-phospho-(1′-*rac*-glycerol) monosodium salt (POPG) were purchased from Avanti Polar Lipids, Inc. (Alabaster, AL). Dipalmitate (DP) was purchased from Sigma-Aldrich Japan Co., LLC. (Tokyo, Japan). Tristearin (TS) and gum arabic were purchased from Wako Pure Chemical Industries. Olive oil was purchased from Nacalai Tesque Inc. (Kyoto, Japan). TOYOPEARL Butyl-650M was from Tosoh Co. (Tokyo, Japan), and Superdex 200 10/300 GL, HiTrap Q HP, HiTrap Phenyl HP, and HisTrap HP columns were from GE Healthcare (Tokyo, Japan). All other chemicals were of the highest available or analytical grade.

### Microbial strains and culture conditions

*C. kessleri* strain 11h (UTEX #263) was purchased from the Culture Collection of Algae at the University of Texas at Austin (UTEX). Sixteen milliliters of *C. kessleri* seed culture were transferred into a 1-L bottle containing 0.8 L of 0.2 × B5 culture medium (Gamborg et al. 1968) and cultivated at 30 °C under continuous white fluorescent light irradiation of 7000 lx with bubbling of 500 mL min^−1^ air containing 3% (*v*/*v*) CO_2_. Cells were subcultured every 7 days by making 1:50 dilutions in fresh 0.2 × B5 medium. To use cells, cultures were harvested after 14 days.

*E. coli* JM109 (Takara Bio., Shiga, Japan) was commonly used as the host strain for molecular cloning and strain BL21 (Novagen, Madison, WI) was employed for functional expression of ckGL. Plasmids pUC118 and pCold-TF (Takara Bio) were used as the cloning and expression vector, respectively. *E. coli* cells were usually cultured in Luria-Bertani (LB) medium at 37 °C, and the medium was supplemented with 100 μg mL^−1^ of ampicillin, unless otherwise indicated.

### Enzyme purification

All purification procedures were performed at 4 °C. Strain 11h cells cultured in 2-L medium were obtained by centrifugation (2400×*g* for 5 min), and the cell pellet (33.3 wet-g of cells) was suspended in 50 mL of 20 mM Tris-HCl buffer, pH 8.0 (buffer A), followed by disruption using a BeadBeater (Biospec Products, Bartlesville, OK). The resultant lysate was centrifuged (2400×*g* for 5 min), Triton X-100 was added to a final concentration of 1% (*w*/*v*), and the mixture was solubilized by stirring for 2 h. The sample was centrifuged (210,000×*g* for 1 h), and the supernatant was collected as the cell-free extract (cfe, 130 mL). After addition of three volumes of cold acetone (− 20 °C), the solution was stood at − 20 °C overnight. Then, the sample was centrifuged (12,000×*g* for 10 min) and the pellet was suspended in 150 mL of buffer A containing 2 M KCl, 1% (*w*/*v*) Triton X-100 and 1 mM dithiothreitol (DTT) (buffer B). After centrifugation (20,000×*g* for 30 min), the resulting supernatant was loaded onto a Butyl-650 M column (2.5 × 8.5 cm) equilibrated with buffer B. The column was washed with three column volumes (CV) of buffer B, and proteins were eluted with a linear gradient (five CV) of 2–0 M KCl in 1% (*w*/*v*) Triton X-100/buffer A at 10 mL min^−1^. The active fractions were pooled and dialyzed against 1% (*w*/*v*) Triton X-100/buffer A, after which the sample was applied to a HiTrap DEAE FF column (5 mL) equilibrated with the same buffer. After washing the column (three CV), the proteins were eluted with a linear gradient (10 CV) of 0–1 M NaCl in 1% (*w*/*v*) Triton X-100/buffer A at 5 mL min^−1^. The active fractions were pooled, and the buffer was exchanged with buffer B using a Vivaspin 20 centrifugal concentrator 10,000 MWCO (Vivaspin 20-10 k; GE Healthcare Japan), followed by application to a HiTrap Butyl HP column (5 mL) equilibrated with buffer B. After washing the column (three CV), the proteins were eluted with a linear gradient (16 CV) of 2–0 M KCl in buffer B at 2 mL min^−1^. Fractions exhibiting high specific GL activity and displaying a single band on sodium dodecyl sulfate-polyacrylamide gel electrophoresis (SDS-PAGE) were pooled, and then the buffer was exchanged with 1% (*w*/*v*) Triton X-100/buffer A using a Vivaspin 20. This protein was used for subsequent investigation.

### Standard assay for enzyme activity

A reaction mixture (22.5 μL), containing 15 μL of 0.2 M MES-NaOH buffer (pH 6.5), 2.5 μL of 1% (*w*/*v*) DGDG/1% (*w*/*v*) Triton X-100, 2.5 μL of 1% (*w*/*v*) Triton X-100, and 2.5 μL of 20 mM CaCl_2_ was incubated at 37 °C for 5 min. Thereafter, 2.5 μL of enzyme solution containing 1% (*w*/*v*) Triton X-100/buffer A was added to the reaction mixture and incubated at 37 °C for an additional 5 min. The reaction was stopped by incubation at 100 °C for 5 min. After centrifugation (21,800×*g* for 5 min), the concentrations of FFAs released by the enzyme reaction were determined with a NEFA C Kit (Wako Pure Chemical Industries) using oleic acid as a standard and in accordance with the manufacturer’s instructions. The concentration of FFAs produced was determined by measuring the absorbance at 550 nm (A_550_). One unit (U) of enzyme activity was defined as the amount of enzyme that catalyzed the formation of 1 μmol of FFAs per min.

### Effect of pH, temperature, and chemicals on enzyme activity

Six buffers (sodium acetate, MES-NaOH, Bis-Tris-HCl, HEPES-NaOH, Tris-HCl, and glycine-NaOH) were used to identify the pH at which the enzyme activity toward DGDG was maximal and to determine the pH stability of the purified enzyme. The optimum pH was determined by incubation at 37 °C for 5 min in 0.14 M of each buffer containing 0.3% (*w*/*v*) Triton X-100 and 2 mM CaCl_2_. The pH stability was assayed by incubating the purified enzyme at 4 °C for 3 h in 50 mM of each buffer. The residual activity was assayed in the standard assay conditions. To identify the temperature at which activity toward DGDG was maximal, the enzyme activity was determined at selected temperatures in the standard assay conditions. Thermal stability was determined by incubating the purified enzyme in 0.14 M MES-NaOH buffer (pH 6.5) for 30 min at each temperature, after which, the residual activity was measured by incubation in the standard assay conditions. To determine the effect of chemicals such as metal ions and EDTA on the enzyme activity, the purified enzyme was assayed in the standard assay conditions in the presence of 2 mM chemicals (+ 10 mM CaCl_2_) or 10 mM metal ions. Inhibitors assessed were EDTA, 2-mercaptoethanol (2-ME), DTT, iodoacetamide (IAA), and phenylmethanesulfonyl fluoride (PMSF).

### Substrate specificity

The substrate specificity of the purified enzyme was analyzed by assaying enzyme activity in the optimum reaction conditions (37 °C and pH 6.5) in the presence of 2 mM Ca^2+^ and 0.3% (*w*/*v*) Triton X-100, using galactolipid or phospholipid substrates. For DP, TS, and olive oil, 0.2 g of acylglycerolipid substrate was emulsified with 10 mL distilled water and 0.1 g of gum arabic using a sonicator (TOMY UD-211). The enzyme activity was determined in the standard assay conditions using the emulsified substrate.

### Protein analysis

Protein concentrations were determined using a Pierce BCA protein assay kit (Thermo Fisher Scientific K.K., Yokohama, Japan) with BSA as the standard. Protein samples were analyzed by SDS-PAGE using the method of Laemmli (Laemmli 1970). Gels were stained with Coomassie Brilliant Blue R-250 (CBB), Simply Blue Safe Stain (Thermo Fisher Scientific), or periodate-Schiff reagent (Irvin et al. 1984). The molecular mass of the purified enzyme was estimated by gel filtration using a Superdex 200 10/300 GL column with 0.15 M NaCl, 1% (*w*/*v*) Triton X-100/20 mM Tris-HCl buffer (pH 7.5), and by native- or blue native-PAGE (BN-PAGE; Life Technologies Corporation, Carlsbad, CA).

### Peptide sequencing

The purified enzyme was resolved using SDS-PAGE and then electroblotted onto polyvinylidene difluoride membrane (Immobilon-P^SQ^ transfer membrane, Millipore Co., Billerica, MA), which was stained with CBB. The transferred 53 kDa band was excised and subjected to *N*-terminal amino acid (aa) sequence analysis (Procise 494 HT Protein Sequencing System; Applied Biosystems, Foster City, CA). For internal aa sequencing, the protein band was excised and decolorized using 30% acetonitrile containing 25 mM (NH_4_)_2_HCO_3_, after which, in-gel digestion was performed using the method described by Shevchenko et al. (Shevchenko et al. 1996). Briefly, the excised protein band was digested with trypsin (Sequencing Grade Modified Trypsin, Promega Corporation, Madison, WI) for 45 h at 4 °C, after which, a sample (1 μL) of the extracted peptides was separated using a nanoAcquity UPLC system (Waters Corp., Milford, MA) equipped with a nanoAcquity UPLC^®^ BEH130 C18 column (Waters Corp., 75 μm × 150 mm, 1.7 μm), and then analyzed using a Xevo QTOF MS as previously described (Sugimori et al. 2012). De novo sequencing of each peptide was performed using the ProteinLynx Global SERVER, version 2.3 (Waters Corp.).

### Gene analysis and cDNA cloning

*C. kessleri* cells were ground in liquid nitrogen using a mortar and pestle, and genomic DNA was extracted from the fractured cells using NucleoSpin Plant II (Macherey-Nagel, Düren, Germany). Then, a Nextera DNA sample Preparation Kit (Illumina, Little Chesterford, UK) was used to generate a sequencing library of 50 ng of the genomic DNA according to the manufacturer’s instructions. The DNA library was purified and simultaneously size-selected by using a DNA Clean & Concentration Kit-5 (Zymo Research, CA) and Agencourt AMPure XP beads (Beckman-Coulter, CA). After the 2 × 250 bp MiSeq paired-end sequencing run, the sequence reads obtained were assembled using the de novo assembly program in CLC Genomics Workbench software (CLC Bio, Aarhus, Denmark) to obtain a draft sequence of the genome. Finally, the genomic sequence of *glp1* was confirmed by Sanger sequencing of a DNA fragment amplified by PCR using primers, 5′-acccgcaaagcagtgggtcaagaactcag-3′ and 5′-caattgggcctcagctccacaggaaagg-3′.

Total RNA was extracted from *C. kessleri* cells using RNeasy mini (Qiagen, Hilden, Germany) according to the manufacturer’s instructions. First-round PCR for cDNA synthesis was performed by using a SMART RACE cDNA Amplification Kit (Takara Bio) and the synthesized DNA fragments were used as templates for the second round of PCR. The 5′- and 3′- sequences of the *glp1* cDNA were obtained by 5′- and 3′- rapid amplification of cDNA ends (5′- and 3′-RACE) with the gene specific primers 5′-cttccactcgccgcccgtgatggtg-3′ for 5′-RACE, and 5′-tctgaagtcagtgaggctcggcag-3′ for 3′-RACE. These primers were designed based on internal (GTITGGEWK) and *N*-terminal (TALREVATKSEVSEA) aa sequences of ckGL and on the nucleotide sequence of the *C. kessleri* genome revealed by the de novo sequencing described above.

The second round of PCR was performed using the nested universal primer (5′-aagcagtggtatcaacgcagagt-3′) provided in the kit, along with the gene-specific primers employed for 5′-RACE and 3′-RACE, mentioned above. The second-round PCR reaction mixture (50 μL) contained 1× PCR Buffer, 10 pmol of each primer pair, 1 U of KOD FX Neo polymerase (Toyobo Co., Ltd.), and 1 μL of the first cDNA reaction mixture as the template. The thermal cycling parameters were 94 °C for 2 min, followed by 40 cycles of 98 °C for 10 s, and 68 °C for 2 min. After the PCR amplification, the two PCR fragments obtained were cloned into pUC118 by using a Mighty Cloning Reagent Set (Takara Bio). Sequencing of the two partial regions of the cDNA fragment of *glp1* encoding ckGL was performed with a BigDye Terminator Cycle Sequencing Kit and analyzed in a 3130 Genetic Analyzer (Applied Biosystems).

For cloning of *glp1*-cDNA, gene-specific forward (5′-atgcgccgtgcttctttggcaac-3′) and reverse (5′-tcacttgccataggtatctttc-3′) primers were designed. PCR was then performed using 10 pmol of the gene-specific primers and 1 μL of each of the first cDNA reaction mixtures as the templates. The thermal cycling parameters were 94 °C for 2 min, followed by 40 cycles of 98 °C for 10 s, and 68 °C for 2 min. The PCR fragments obtained were cloned into pUC118 by using a Mighty Cloning Reagent Set and finally pckGL1, containing *glp1*-cDNA, was constructed.

### Expression of recombinant ckGL in *E. coli*

The recombinant ckGL (rckGL) expression plasmid pCold TF-ckGL1 comprising DNA regions coding for a His-tag, TF (trigger factor), factor Xa recognition site, and mature ckGL was constructed based on vector pCold TF (Takara Bio) as follows (Fig. S1). The DNA fragment encoding mature ckGL was amplified using primers 5′-ggtatcgaaggtaggactgccctgcgcgaggtt-3′ (*N*-terminal In-Fusion cloning site, underlined) and 5′-agcagagattacctatcacttgccataggtatctt-3′ (*C*-terminal In-Fusion cloning site, underlined) with *glp1*-cDNA as the template, and a DNA fragment of the vector was amplified with primers 5′-taggtaatctctgcttaaaagcac-3′ (*C*-terminal In-Fusion site, underlined) and 5′-cctaccttcgataccaccactac-3′ (*N*-terminal In-Fusion site, underlined) using pCold TF as the template. The PCR reaction mixture (50 μL) contained 1× PCR Buffer, 10 pmol of each primer set, 20 nmol dNTPs, 1 U of KOD FX Neo polymerase, and 5 ng of pckGL1 and pCold TF, respectively. The PCR conditions were: an initial denaturation step at 94 °C for 2 min, followed by 35 cycles of amplification at 98 °C for 10 s, and 68 °C for 1 min per kb. Then, these PCR products were purified using the MinElute PCR Purification Kit (QIAGEN) and digested with *Dpn*I (Takara Bio) to remove the original template plasmid DNA. An In-Fusion HD cloning kit (Takara Bio) was used to fuse the desired PCR products and then the fusion products were transformed into *E. coli* JM109 competent cells to clone the pCold TF-ckGL1 plasmid. Transformants (JM109/pCold TF-ckGL1) were selected by ampicillin resistance on LB agar plates.

*E. coli* BL21 cells carrying pCold TF-ckGL1 were inoculated into a test tube containing LB seed medium and ampicillin and cultivated overnight at 37 °C with shaking. Thereafter, 1% (*v*/*v*) inoculum was transferred to 5 mL of LB medium supplemented with 100 μg mL^−1^ ampicillin. When the optical density of the culture at 660 nm reached ~ 0.5, the culture temperature was shifted to 15 °C and isopropyl β-D-1-thiogalactopyranoside was added to a final concentration of 0.1 mM. After low-temperature incubation at 15 °C for an additional 24 h, the cell culture (5 mL × 10) was centrifuged (18,800×*g* for 5 min), and the resulting cell paste (0.11 g-wet cells) was washed with 1% (*w*/*v*) Triton X-100/buffer A (buffer T) and resuspended in 19 mL of the same buffer T. The cells were sonicated with a TOMY UR-20P (28 kHz, 10 W, 18 min, 4 °C), and the suspension was centrifuged at 21,600×*g* for 10 min. The supernatant (cfe) of the cell lysate was collected and applied to a HisTrap HP column (5 mL) equilibrated with 5 mM imidazole, 0.5 M NaCl/buffer T (pH 7.5). After washing the column (three CV), the proteins were eluted with a linear gradient (20 CV) of 5–500 mM imidazole in buffer T at 5 mL min^−1^. The active fractions were pooled, and the buffer was exchanged with buffer A using a Vivaspin 20–30,000 MWCO. The purified His-tag fused TF-ckGL sample was incubated at 7 °C for 24 h in 2 mM CaCl_2_, 0.1 M NaCl/50 mM buffer A, and 3 mU factor Xa (New England Biolabs Japan Inc., Tokyo), after which it was applied to a HisTrap HP column (1 mL) equilibrated with 5 mM imidazole and 0.5 M NaCl/buffer T (pH 7.5). This step separated the His-tag-TF portion and protein retaining the His-tag-TF portion from rckGL protein. The elution method was the same as above, at 1 mL min^−1^. Fractions exhibiting high specific GL activity and displaying a single band on SDS-PAGE analysis were pooled, and then the buffer was exchanged with buffer T using a Vivaspin 20-10 k. This protein was used for subsequent investigation.

### Nucleotide and peptide sequence accession number

The nucleotide sequences of the *glp1*-cDNA for ckGL and *glp1* were deposited in the DDBJ/GenBank/EMBL database under accession numbers LC094143 and LC314400, respectively.

### Steady-state kinetics

For the purified native ckGL enzyme, the initial velocity (*v*) of the enzymatic reaction was determined at several DGDG concentrations ([DGDG]) in the standard assay conditions (37 °C, pH 6.5). The concentration of the enzyme in the reaction mixture was held constant at 0.419 μg L^−1^ (7.93 nM), calculated as monomeric mature protein with a subunit molecular mass of 52,802 Da. The corresponding 1/*v* vs. 1/[DGDG] plot was evaluated using the Michaelis-Menten equation. Kinetic constants *K*_m_, *V*_max_, and *k*_cat_ were determined using linear regression (KaleidaGraph, Synergy Software, Reading, PA). *v* of the enzymatic reaction was also determined as described above for rckGL. The concentration of the purified rckGL in the reaction mixture was held constant at 1.50 μg mL^−1^ (28.2 nM). Kinetic constants *K*_m_, *V*_max_, and *k*_cat_ were determined using nonlinear regression (KaleidaGraph).

### Gas chromatography analysis

The positional specificity of the hydrolytic reaction was determined by capillary gas chromatography (GC) analysis using purified rckGL. The enzymatic reaction (100 μL) containing 0.1% DGDG or POPG, 2 mM Ca^2+^, and 0.3% (*w*/*v*) Triton X-100 was performed in the optimum assay conditions (37 °C and pH 6.5). Samples (25 μL) were withdrawn from the reaction mixture 2, 3, 5, and 15 min after the start of the reaction. The enzyme reaction was terminated immediately by extracting with chloroform-methanol (2:1, *v*/*v*). One microliter of the extract was injected with a split ratio of 50:1 into a Shimadzu GC-14B (Kyoto, Japan) chromatograph system equipped with a Nukol column (15 m × 0.53 mm × 0.50 μm; Sigma-Aldrich). The GC operation conditions were: the GC column was heated at 8 °C min^−1^ from 110 to 220 °C and held for 15 min at 220 °C; the injector and detector temperature was 250 °C; and the flow rate of the He carrier gas was 25 mL min^−1^. The released FFAs were separated, and then the concentrations were determined by using oleic acid (18:1), palmitic acid (16:0), and linolenic acid (18:3) of known concentrations as standards.

## Results

### Purification of ckGL

**Fig. 2:**
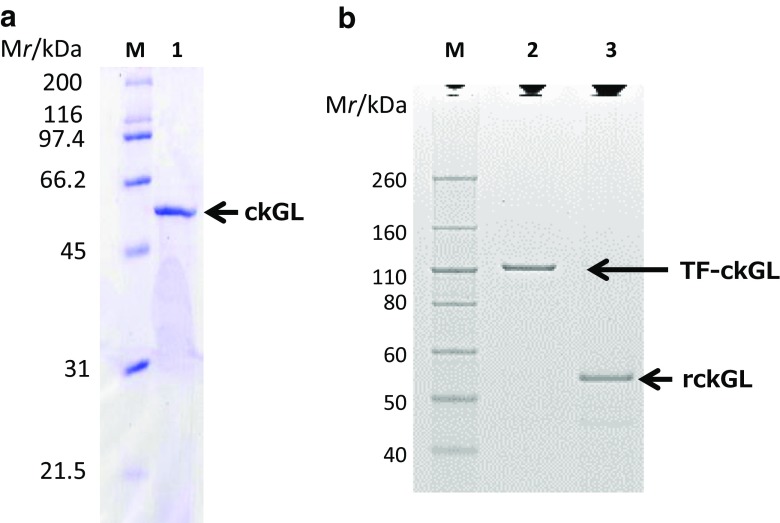
SDS-PAGE analysis of **a** the purified native ckGL from *C. kessleri* and **b** recombinant ckGL (rckGL) produced in *E. coli* BL21*.* Lane M, protein molecular mass markers; lane 1, purified enzyme (2.9 μg protein); lane 2, recombinant enzyme in TF-ckGL form (0.1 μg); lane 3, rckGL (0.1 μg) produced from TF-ckGL by digestion with Factor Xa. The SDS-PAGE gels **a** and **b** were stained with Coomassie Brilliant Blue R-250 and Simply Blue Safe Stain (G-250), respectively

The overall purification of ckGL from culture of strain 11h was 163-fold, with an activity yield of 6.07% (Table 1). It was not possible to purify active enzyme in the absence of Triton X-100 or by using other detergents because the enzyme became inactive during the procedure. The specific activity of the purified enzyme was 143 U mg-protein^−1^ and the final yield was 0.557 mg of protein. When the purified enzyme was subjected to SDS-PAGE, a single band with an apparent molecular mass of ~ 53 kDa was observed upon CBB staining (Fig. 2a). The purified protein was not stained by periodate-Schiff reagent, however, suggesting that the enzyme is not a glycoprotein. In gel-filtration chromatography, the enzyme was eluted in the void volume, and a smear band (146–242 kDa) was observed on native- and BN-PAGE. The *N*-terminal aa sequence was determined to be principally TALREVATKSEVSEA (Fig. S2). A minor fraction (accounting for 20–30%) had sequence ATALRE, which was presumed to be an additional alanyl-form of the protein. In addition, nine peptide sequences were detected as internal aa sequences of ckGL by LC-MS/MS analysis (Fig. S2), and they were used for construction of PCR primers for ckGL gene (*glp1*) cloning.

**Table 1:** Purification of GL from *Chlorella kessleri*

Purification step	Sample (mL)	Total protein (mg)	Specific activity^a^ (U/mg of protein)	Total activity (U)	Purification (fold)	% Recovery
Cell-free extract	130	1495	0.878	1313	1	100
Acetone precipitation	150	546	0.514	281	0.585	21.4
Butyl-650 M	172	14.9	31.4	466	35.8	35.5
HiTrap DEAE FF	10.7	0.492	127	62.3	145	4.74
HiTrap Butyl HP	19.2	0.557	143	79.7	163	6.07

^a^Enzyme activity toward DGDG was assayed in 0.14 M Tris-HCl buffer (pH 7.2), 0.1% (*w*/*v*) DGDG, 0.2% (*w*/*v*) Triton X-100, and 10 mM CaCl_2_ (37 °C for 5 min)

### Enzymatic characterization of ckGL

**Fig. 3:**
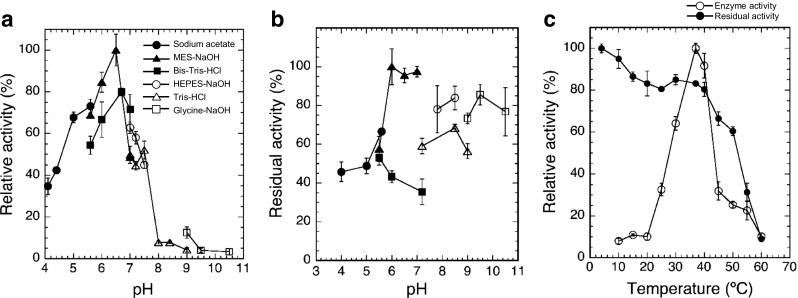
Effect of pH and temperature on DGDG hydrolytic activity and stability of ckGL. **a** Effect of pH on ckGL activity. The enzyme activity was assayed at 37 °C for 5 min in 0.14 M of each buffer containing 0.3% (*w*/*v*) Triton X-100 and 2 mM CaCl_2_. The buffers were: sodium acetate (pH 4.1–5.6), MES-NaOH (pH 5.6–7.0), Bis-Tris-HCl (pH 5.6–7.0), HEPES-NaOH (pH 7.0–7.5), Tris-HCl (pH 7.0–9.0), and glycine-NaOH (pH 9.0–10.5). **b** The pH stability of ckGL. The residual activity after incubating at 4 °C for 3 h in 50 mM of each buffer. The buffers used were the same as above. The residual activity was assayed at 37 °C for 5 min in the standard conditions, i.e., 0.14 M MES-NaOH buffer (pH 6.5) containing 0.3% (*w*/*v*) Triton X-100 and 2 mM CaCl_2_. **c** The effect of temperature on enzyme activity and stability. The enzyme activity was assayed at each temperature for 5 min in 0.14 M MES-NaOH buffer (pH 6.5). The residual activity was assayed in the standard conditions after incubating at each temperature for 30 min in 0.14 M MES-NaOH buffer (pH 6.5). Data are the means of experiments performed in triplicate. Error bars represent the standard deviation

**Fig. 4:**
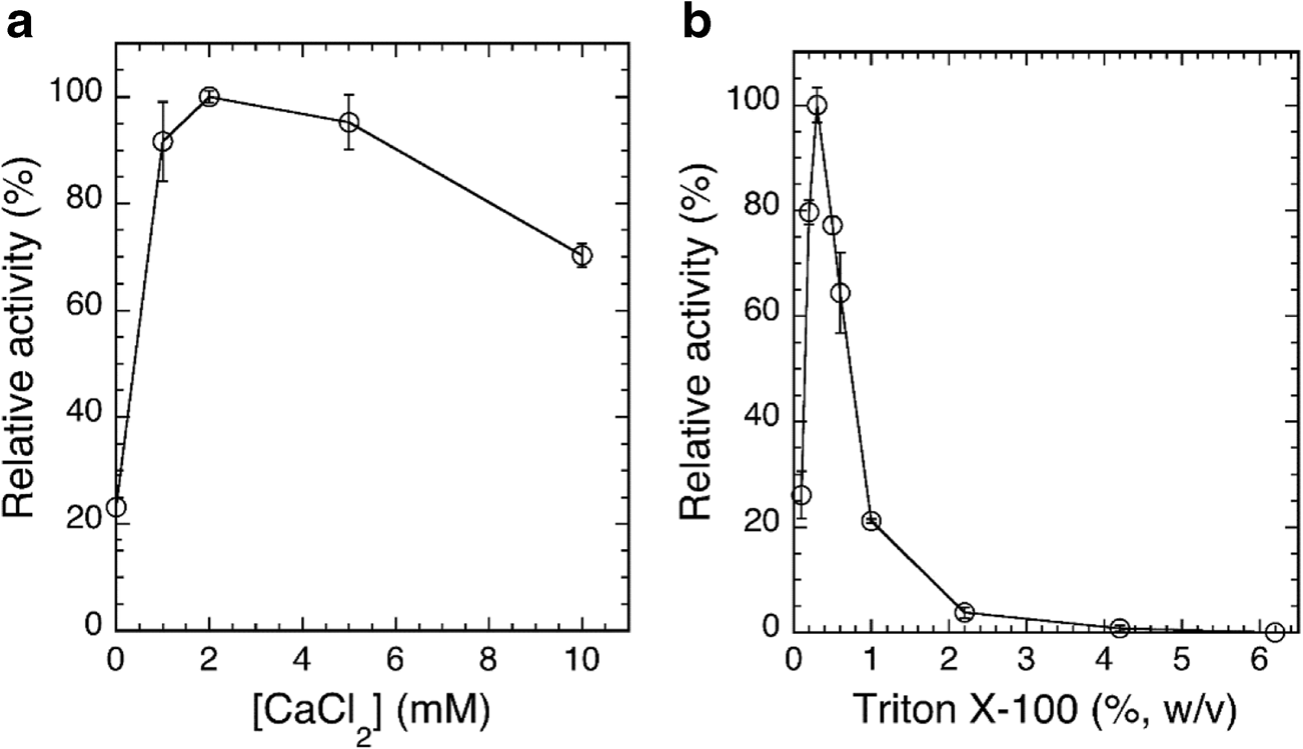
Effects of CaCl_2_ or Triton X-100 concentration in the reaction mixture on ckGL activity. **a** The enzyme activity was assayed at 37 °C for 5 min in 0.14 M MES-NaOH buffer (pH 6.5) and 0.2% (*w*/*v*) Triton X-100 in the presence of 0–10 mM CaCl_2_. **b** The enzyme activity was assayed at 37 °C in 0.14 M MES-NaOH (pH 6.5) and 2 mM CaCl_2_ in the presence of 0.1–6.2% (*w*/*v*) Triton X-100. Data are the means of experiments performed in triplicate. Error bars represent the standard deviation

The highest activity for DGDG hydrolysis by purified ckGL was detected at around pH 6.5 and 37 °C (Fig. 3a, c). The enzyme was stable from pH 6 to pH 7 and from 4 to 40 °C (Fig. 3b, c). An Arrhenius plot showed an activation energy (*E*_*a*_) for DGDG of 82.9 kJ mol^−1^ and a preexponential factor (*A*) of 6.50 × 10^16^ (s^−1^). ckGL was strongly activated by Ca^2+^ and considerably activated by Mn^2+^, and the maximal activity was detected in the presence of 2 mM Ca^2+^ (Fig. 4a). The enzyme was strongly inhibited by Zn^2+^, Fe^2+/3+^, and EDTA (Table 2), while IAA, 2-ME, DTT, PMSF, and SDS modestly inhibited the enzyme activity. Moreover, Triton X-100 strongly stimulated the enzyme activity, and the absence of this detergent caused the inactivation of ckGL even in the enzyme assay, as it did during the purification. Thus, the maximal activity was detected in the presence of 0.3% (*w*/*v*) Triton X-100 (Fig. 4b).

**Table 2:** Effect of metal ions and chemicals on activity of ckGL in DGDG hydrolysis

Chemical	Relative activity (%)^a^
Control (Ca-free)	29.4 ± 0.4
10 mM CaCl_2_	100 ± 7
10 mM MnCl_2_	86.1 ± 3.1
10 mM MgCl_2_	38.2 ± 1.5
10 mM ZnCl_2_	0
10 mM FeCl_2_	0
10 mM FeCl_3_	2.39 ± 2.51
2 mM EDTA	10.3 ± 5.1
2 mM SDS^b^	69.9 ± 3.6
2 mM IAA^b^	43.8 ± 5.1
2 mM 2-ME^b^	72.6 ± 3.2
2 mM DTT^b^	56.2 ± 14.6
2 mM PMSF^b^	66.2 ± 14.9

^a^The enzyme activity was assayed using a reaction mixture containing 0.14 M MES-NaOH buffer (pH 6.5), 0.3% (*w*/*v*) Triton X-100, and 10 mM metal ion or 2 mM chemical at 37 °C. The relative activity was determined by defining the activity in the presence of 10 mM CaCl_2_ as 100%

^b^For chemicals except for EDTA, reaction mixture containing 10 mM CaCl_2_ was used for the assay. Data represent the means and standard deviations of experiments performed in triplicate

**Fig. 5:**
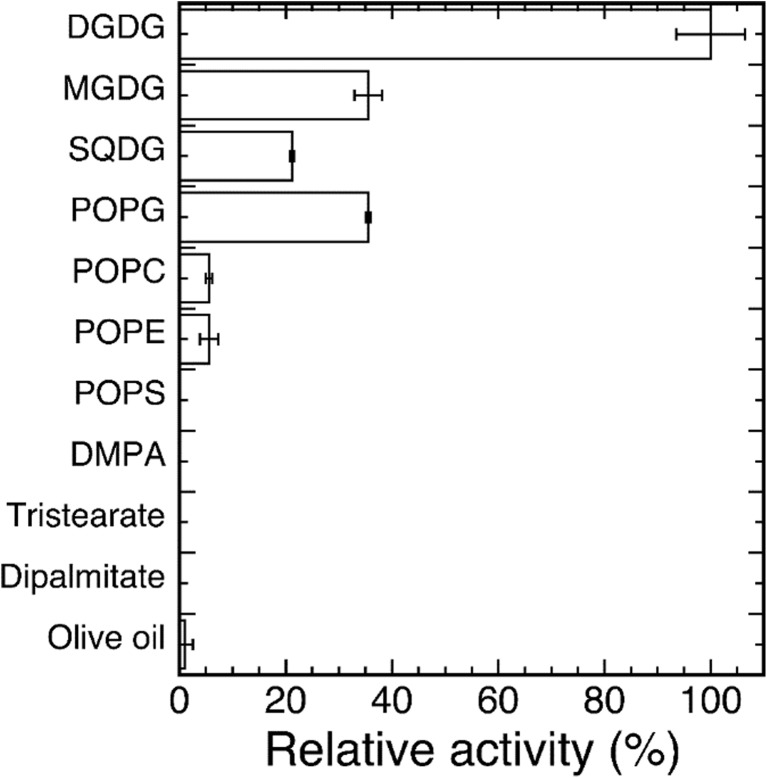
Substrate specificity of ckGL. The enzyme activity toward each substrate was assayed in the standard conditions: 0.14 M MES-NaOH buffer (pH 6.5) containing 0.3% (*w*/*v*) Triton X-100 and 2 mM CaCl_2_. Data are the means of experiments performed in triplicate. Error bars represent the standard deviation

The highest activity of ckGL was toward DGDG as the substrate, and the activities toward MGDG, SQDG, and POPG were moderate (Fig. 5). ckGL exhibited almost no activity toward glycerides and phospholipids such as TS and PC.

The apparent *V*_max_ and *k*_cat_ values for DGDG hydrolysis by native ckGL were 572 μmol min^−1^ mg-protein^−1^ (239 μΜ min^−1^) and 498 ± 112 s^−1^, respectively, and the apparent *K*_m_ and *k*_cat_/*K*_m_ values were 1.05 ± 0.48 mM and 538 ± 142 s^−1^ mM^−1^, respectively (Fig. S3a).

### *glp1* gene and cDNA cloning

We analyzed the genome sequence of *C. kessleri* strain 11h and obtained the draft DNA sequence. Then, based on the partial aa sequences of ckGL obtained experimentally, the *glp1* gene was tentatively localized in the genome sequence, and we acquired authentic nucleotide sequences corresponding to partial regions of ckGL for the purpose of DNA primer design for cDNA cloning. Through 5′- and 3′-RACE after cDNA library construction, the full-length cDNA sequence of *glp1* was identified (Fig. S4). The *glp1* gene (5.6 kbp) was composed of 10 introns and 11 exons, all of which adhere to the “gt-ag” rule of eukaryotic splicing (Padgett et al. 1986). The 1608-bp full-length cDNA encoded a mature ckGL comprising 475 aa residues, with a presequence of 60 aa containing a potential chloroplast transit peptide (cTP) (Fig. S2). An *N*-terminal cTP directs the protein into the target organelle (Emanuelsson et al. 1999) and is subsequently removed by proteases. We analyzed the deduced aa sequence of ckGL using the ChloroP v1.1 prediction program (http://www.cbs.dtu.dk/services/ChloroP), and it predicted that the *N*-terminal 59 aa residues of premature ckGL are a potential cTP (Fig. S2). Since we identified a Thr residue at position 61 as the predominant *N*-terminal aa of mature ckGL, the Ala residue at position 60 (Ala60) might be removed by an aminopeptidase after the transport of ckGL into the chloroplast. Thus, we concluded that the mature form of ckGL comprises 475 aa residues (Fig. S2). The molecular weight of the gene product (i.e., the 475 aa mature ckGL) was calculated to be 52,801, which is consistent with the molecular weight of the purified enzyme estimated from SDS-PAGE. The isoelectric point (pI) of ckGL was calculated to be 6.32 using Genetyx-Mac version 17.0.5 (Genetyx Corporation). In addition, the internal aa sequences of ckGL were all assigned to the sequence deduced from the *glp1*-cDNA (Fig. S2). Therefore, these results indicated that the obtained cDNA actually encodes ckGL.

### Functional expression and purification of recombinant ckGL

Functional production of rckGL was achieved in *E. coli* BL21 cells transformed with the expression plasmid pCold TF-ckGL1 (Fig. S1). From 50 mL cell culture of the transformed *E. coli* cells, we obtained 0.11 g of wet cell paste, and then the cfe with DGDG hydrolytic activity of 2.18 U mL^−1^ was prepared, indicating that His-tag-TF fused rckGL is an active form of the enzyme. The specific activity of the cfe (4.86 U mg-protein^−1^) was ~ 6 fold higher than that in the cfe prepared from *C. kessleri* (0.878 U mg-protein^−1^). Then, rckGL (92.1 μg-protein) with high specific activity (75.2 U mg-protein^−1^) and total activity (6.93 U) was purified to electrophoretic homogeneity (Fig. 2b) from the cfe (19 mL) by simple purification steps including cleavage of the His-tag and TF (Table 3).

**Table 3:** Purification of rckGL

Purification step	Sample (mL)	Total protein (mg)	Specific activity^a^ (U/mg of protein)	Total activity (U)	Purification (fold)	% Recovery
Cell-free extract	19.0	8.51	4.86	41.4	1.00	100
1st HisTrap HP (5 mL)	18.4	1.98	19.9	39.5	4.10	95.4
2nd HisTrap HP (1 mL)	3.08	0.0921	75.2	6.93	15.5	16.8

^a^rckGL activity toward DGDG was assayed in the standard assay conditions (pH 6.5, 37 °C) containing 0.3% (*w*/*v*) Triton X-100 and 2 mM CaCl_2_

Subsequently, as for native ckGL, the kinetic parameters of rckGL were analyzed. For DGDG hydrolysis by rckGL, the apparent *V*_max_ and *k*_cat_ values were 1.57 mmol min^−1^ mg-protein^−1^ (2.34 mΜ min^−1^) and 1390 ± 153 s^−1^, respectively, and the apparent *K*_m_ and *k*_cat_/*K*_m_ values were 0.54 ± 0.11 mM and 2622 ± 223 s^−1^ mM^−1^, respectively (Fig. S3b).

**Fig. 6:**
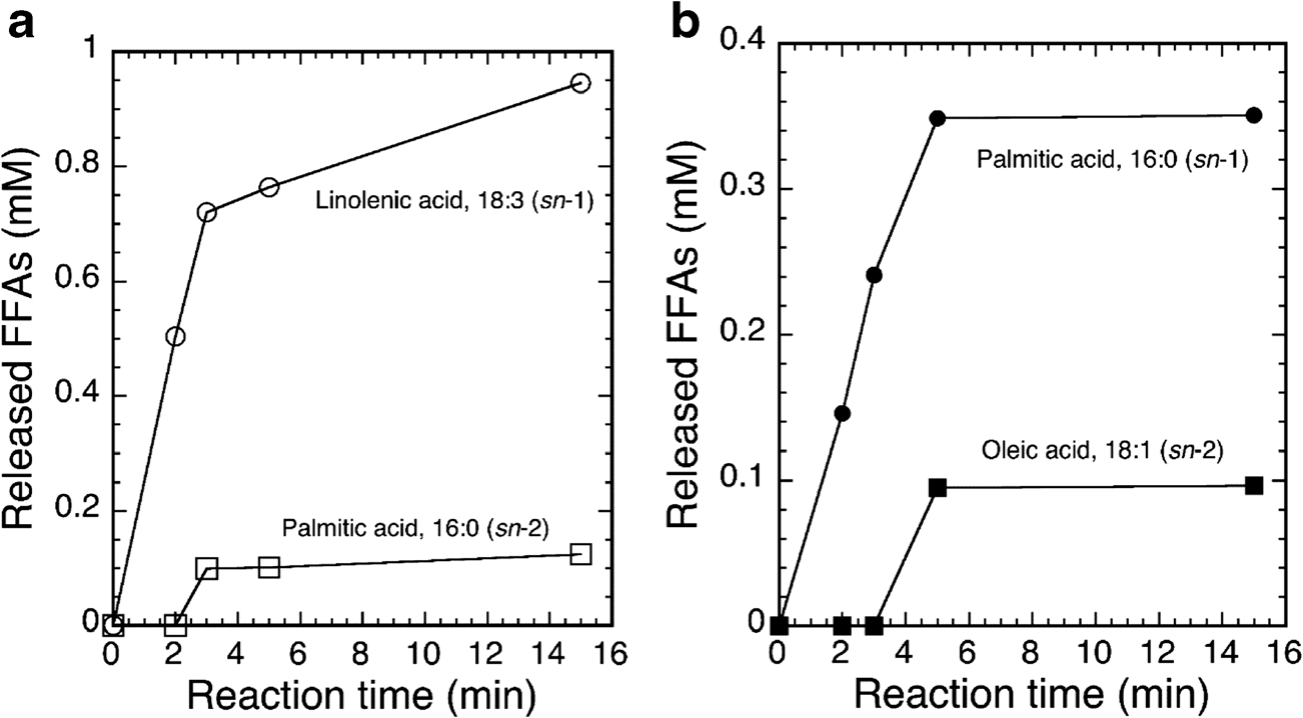
Time course of GC analysis of DGDG (**a**) and 1-palmitoyl-2-oleoyl-*sn*-glycero-3-phospho-(1′-*rac*-glycerol) monosodium salt (POPG) (**b**) hydrolysis by rckGL. The enzyme reaction was carried out in the standard conditions. The concentrations of released FFAs were determined by using oleic acid (18:1), palmitic acid (16:0), and linolenic acid (18:3) of known concentrations as standards. Data shown are from a typical experiment

In addition, we analyzed the hydrolytic site specificity of the acyl ester bond of the substrate cleaved by the action of rckGL. As Fig. 6 shows, GC analysis indicated that rckGL predominantly hydrolyzed the *sn*-1 acyl ester bond of DGDG as well as POPG because of rapid release of linolenic acid (C_18:3_ fatty acid) from DGDG and palmitic acid (C_16:0_ fatty acid) from POPG, respectively. Since, it has been well known that the vast majority of natural DGDG molecular species contain a C_18_ fatty acid at the *sn*-1 position and a C_16_ fatty acid at the *sn*-2 position (Cho and Thompson Jr. 1987). In fact, DGDG from Avanti Polar Lipids, Inc. contains 44.5% of linolenic acid at the *sn*-1 position.

### Comparative sequence analysis of ckGL

**Fig. 7:**
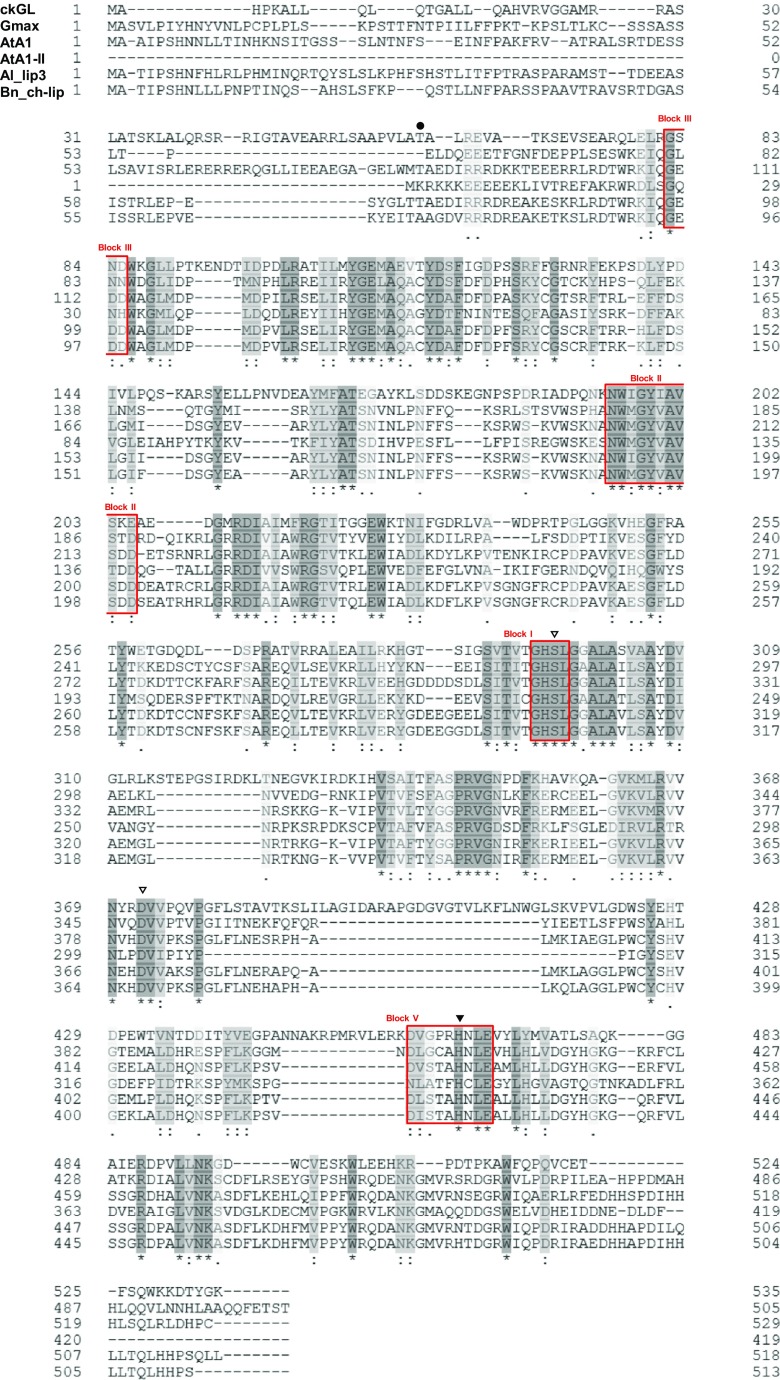
Sequence alignment of the deduced aa sequences of ckGL and known GLs, lipases, and PLA_1_s. The alignment was performed using Clustal Omega program of UniProt. ckGL, the premature ckGL protein (the *N*-terminal residue of mature ckGL, Thr61, is indicated with dot); Gmax, uncharacterized protein from *G. max* (UniProtKB accession number I1LIJ8); AtA1, chloroplastic PLA_1_–Iγ2 from *A. thaliana* (Q3EBR6); AtA1-II, PLA_1_–IIγ from *A. thaliana* (O49523); Al_lip3, lipase class 3 family protein from *A. lyrata* subsp. *lyrata* (D7KG67); Bn_ch-lip, chloroplast lipase from *B. napus* (B8Y0L4). Identical and highly conserved aa is shaded in dark and light gray, respectively, for each protein. Symbols: open triangles, putative catalytic residues (Ser296 and Asp372 in ckGL); closed triangle, putative catalytic residue (His465) of ckGL; Blocks I, II, III, V, (in red boxes) consensus sequences of the lipase 3 family

A homology search using the BLAST algorithm of UniProtKB revealed that the aa sequence of mature ckGL shared 31.2, 30.0, 29.4, 30.0, and 28.5% identity (72–79% similarity) with an uncharacterized protein from *Glycine max* (Soybean) (Gmax; UniProt accession no. I1LIJ8), a lipase class 3 family protein from *Arabidopsis lyrata* subsp. *lyrata* (Al_lip3; UniProt accession no. D7KG67), phospholipase A_1_-Iγ2 from *A. thaliana* (AtA1; UniProt accession no. Q3EBR6), phospholipase A_1_-IIγ from *A. thaliana* (AtA1-II; UniProt accession no. O49523), and chloroplast lipase from *Brassica napus* (Bn_ch-lip; UniProt accession no. B8Y0L4), respectively (Fig. 7). It showed only limited similarity with other predicted or uncharacterized phospholipase A_1_ (PLA_1_) sequences. The aa sequence of ckGL also exhibited only low similarity to microbial and microalgal GLs, sharing 12% identity with *Aspergillus japonicus* GL (AjGL; Patent: JP 2008206515-A/6; DDBJ (JPOP) accession no. DD815271) and 20% identity with *C. reinhardtii* GL (CrGL) (Li et al. 2012) (*Chlamydomonas* v5.3 genome in the Phytozome database, http://www.phytozome.net accession number: Cre03.g193500 (*PGD1*)).

Pfam analysis (Punta et al. 2012) showed that ckGL has an α/β hydrolase fold (Ollis et al. 1992) and belongs to the lipase 3 family. In addition, ckGL contains the typical GxSxG motif of serine hydrolases, together with conserved Ser, Asp, and His residues that form the catalytic triad: Ser296, Asp372, and His465 (Fig. 7). Among five consensus sequences (Blocks I–V) of GDSL (motif consensus aa sequence of Gly, Asp, Ser, and Leu around the active site Ser) enzymes (Akoh et al. 2004), four blocks were found in ckGL: ^82^GSND^85^ (Block III), ^195^NWIGYIAVSKE^205^ (Block II), ^294^GHSL^297^ (Block I, GxSxG motif), and ^460^DVGPRHNLE^468^ (Block V) (Fig. 7).

**Fig. 8:**
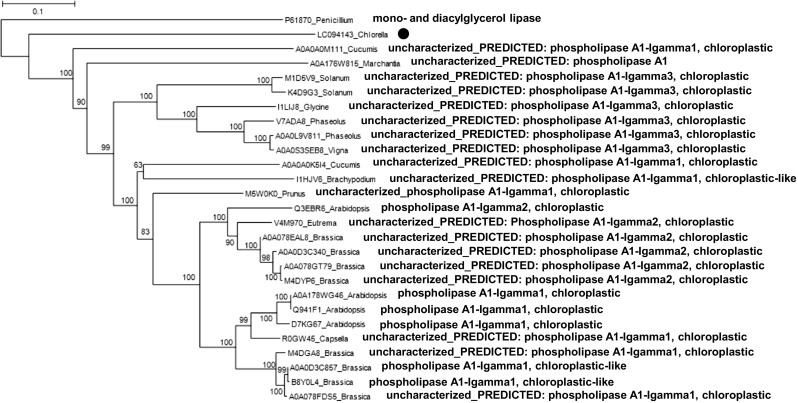
Neighbor-joining phylogenetic tree of ckGL protein (indicated with a dot) and highly homologous proteins in the UniProt database. Numbers at branch nodes are bootstrap percentages based on 1000 replicates. The scale bar indicates an evolutionary distance of 0.1 amino acid residues per position in the sequence. Mono- and diacylglycerol lipase from *Penicillium cyclopium* was employed as the out-group. The phylogenetic comparison of protein sequences was carried out using ClustalW software

A distance-based phylogenetic analysis based on aa sequences also showed that ckGL was clearly separated from groups containing PLA_1_ (Fig. 8), although the aa sequence of ckGL was relatively similar to those of predicted or uncharacterized PLA_1_s derived from chloroplasts in plants including *Cucumis*, *Solanum*, *Brassica*, and *Arabidopsis*. PLA_1_ from *Arabidopsis* chloroplast was experimentally characterized and this acylhydrolase has moderate activity toward DGDG (Seo et al. 2009). Therefore, intriguingly, ckGL of the microalga *Chlorella* may be an ancestor protein of PLA_1_ in the chloroplasts of many plants.

## Discussion

We have identified and characterized a novel GL, a DGDG- and *sn*-1-preferring enzyme, from *Chlorella kessleri*. cDNA for this enzyme (ckGL) was cloned and it was expressed in *E. coli* as an active enzyme. In the cDNA, we found an ORF encoding a 535-aa protein. The predicted initiator Met and its surrounding sequence (5′-ggaaatggc-3′, initiator codon underlined) of *glp1* partially resembled the Kozak consensus sequence (Kozak 1987) and was highly similar to the consensus sequence (5′-aacaatggc-3′) of the translational start region for genes in plants (Lütcke et al. 1987) (Fig. S4). Thus, the first ATG codon in the ORF was assigned as the translational start codon for premature ckGL. Most chloroplast proteins are synthesized inside the cytoplasm as precursors with *N*-terminal extensions (cTPs) and then transferred into the organelle (Keegstra 1989). Although we did not determine the intracellular localization of ckGL, it was predicted that ckGL has a cTP in the *N*-terminal region by a cTP-prediction program (ChloroP). Franzen et al. elucidated that the characteristic features of cTPs from the green alga *C. reinhardtii* are a short uncharged *N*-terminal region, a central region rich in Arg, Ala, and Ser residues with a high propensity for amphiphilic α-helix formation, and a *C*-terminal region forming an amphiphilic β strand (Franźen et al. 1990). In addition, Arg residues are often found in positions − 6 to − 10 from the cleavage site of the cTP, and it was also described that lumen-targeting cTPs from alga frequently contain one negatively charged aa residue (i.e., Asp or Glu), and the aas before the stromal cleavage site (positions of − 3 to − 1) mostly have the sequence Val-Xxx-Ala. The predicted cTP sequence of ckGL contained these structural features (Fig. S2), although the prediction program indicated a cleavage site between the Leu and Ala residues at positions 59 and 60. Since the *N*-terminal aa sequence of purified ckGL was principally TALREV, with ATALREV as a minor fraction, it was supposed that the Ala residue at position 60 would be successively processed by a protease after removal of the cTP region from premature ckGL.

Li et al. produced His-tagged GL (PGD1) from *C. reinhardtii* using *E. coli* strain BL21 (codon+) and expression vector pMK1006; however, the expressed protein was obtained as inclusion bodies (Li et al. 2012). Plant and mammalian GLs of *Vigna unguiculata*, *Cavia porcellus*, and *Homo sapiens* were expressed by baculovirus, *Aspergillus oryzae*, and *Pichia pastoris* systems, respectively (Matos et al. 2000; Hjorth et al. 1993; Eydoux et al. 2007). We successfully produced rckGL in an active form with high specific activity (75.2 U mg-protein^−1^) using the pCold TF fusion expression system. In contrast, direct expression systems using pET or pCold vectors in *E. coli* were not able to produce any active ckGL, suggesting that the TF region might assist the efficient expression or correct folding of the TF-ckGL. TF is a chaperone in *E. coli* and is used for production of fused proteins in a soluble form (Yonath 2006). rckGL was produced in large amounts (~ 40 mg-protein and ~ 800 U per L-culture) as a His_6_-TF-tagged form, while the tag-free form (after in vitro cleavage and purification) was recovered in low amounts. We believe this resulted from factor Xa digestion of the His_6_-TF-tagged form and the subsequent purification via an additional column chromatography step. Since factor Xa digestion required incubation of the rckGL in the reaction mixture overnight, this process certainly resulted in a decrease in rckGL activity.

In contrast, considerable activity and abundance (~ 6% yield) of the native GL enzyme were detected in cell extracts of *C. kessleri*. MGDG and DGDG are mainly used for the membrane lipid in chloroplasts and cyanobacteria instead of phospholipids (Poincelot 1973; Wada and Murata 1989), and several studies have shown that GL activity is associated with the chilling response of plants (Kaniuga 2008). Thus, we consider the high-level activity of ckGL in *C. kessleri* is likely involved in the metabolism of cell- and chloroplast-membrane lipids.

According to the BRENDA database (http://brenda-enzymes.info/enzyme.php?ecno=3.1.1.26), known plant GLs locate to the chloroplast or thylakoid. Although crGL (PGD1) was predicted to be a cytosolic protein, analysis of its subcellular localization using green fluorescent protein fusion constructs was unsuccessful (Li et al. 2012). ckGL was unstable in the absence of Triton X-100, suggesting that it might be membrane-bound or associated. As discussed above, the ChloroP program predicted that ckGL has a cTP, while the SOSUI system and TMHMM server analyses of ckGL suggested that the enzyme is a soluble protein. Therefore, these results imply that ckGL might be located in the chloroplast stroma. Further investigation into the localization of ckGL is required by approaches such as immunolocalization using an antibody.

GLs from cowpea (*V. unguiculata* L.) leaves and the red alga *Gracilaria vermiculophylla* are 40 and 20 kDa homodimers, respectively (Illijas et al. 2008; Sahsah et al. 1994). It was not clear whether ckGL exists in a monomeric or oligomeric form in the cell because of disturbance of the analysis by the need for the presence of Triton X-100 in vitro. The optimum pH for activity of the known GLs is in the range pH 5 to 10.5; ckGL was most active near neutral pH (pH 6.5) in the enzyme reaction, which may result from the pI 6.3 of ckGL. The somewhat broad pH stability may be an intrinsic characteristic of ckGL, but the thermal stability appears lower than that of other known GLs. We concluded that ckGL is a Ca^2+^-dependent enzyme because of the strong activation by Ca^2+^ and inhibition by EDTA. The enzyme was also activated by Mn^2+^, although it was less efficient. Mg^2+^-, Mn^2+^-, and Zn^2+^-dependent GLs were identified from *G. vermiculophylla* (Illijas et al. 2008)*.* Although ckGL contains two Cys residues in the *C*-terminal region (Fig. S2), 2-ME and DTT merely decreased the ckGL activity to moderate levels, thus these Cys residues might not be crucial for the catalytic reaction of ckGL. The substrate specificity of ckGL was remarkably different from that of plant GLs, which show broad substrate specificity toward acylglycerides and phospholipids as well as galactolipids. Moreover, we think the GL family may have functional and genetic diversity from the point of view of aa sequence and substrate specificity. This was supported by phylogenetic analysis indicating that ckGL is evolutionally close to PLA_1_s from many plants (Fig. 8). Apart from the typical motif and characteristic sequences of GDSL enzymes, the deduced aa sequence of ckGL showed little identity with known lipases or crGL (Li et al. 2012) and AjGL.

Li et al. reported that crGL preferred MGDG and the acyl groups at the *sn*-1 position, but did not act on DGDG (Li et al. 2012). In contrast, ckGL preferred DGDG to MGDG and the acyl groups at the *sn*-1 position as the hydrolysis site. The difference in the substrate specificity probably results from the aa sequence and 3D structure of the enzymes. The similarity of the *sn*-1 positional selectivity suggests that the structure and configuration of the substrate-binding pocket may be similar in the two enzymes.

The putative catalytic triad (Ser296, Asp372, and His465) of ckGL is conformationally equivalent to that (Ser236, Asp302, and His339) of PLA_1_-IIγ from *A. thaliana* (AtA1-II; UniProt accession no. O49523, Protein data bank no. 2YIJ), although the position of the His residue of ckGL is comparatively far from that in the other homologs (as well as AtA1-II) in the primary structure. We constructed a modeled structure of ckGL using structural data for AtA1-II, the most suitable template identified by an HHPRED search, as the template (http://toolkit.tuebingen.mpg.de/hhpred) (Söding et al. 2005). The modeled structure of ckGL showed similarity to the catalytic region of AtA1-II; however, the structural surface and overall structure of ckGL were considerably different from those of AtA1-II (Fig. S5). Thus, further investigation into the structure of ckGL is required to elucidate the mechanisms responsible for substrate recognition and to facilitate possible industrial applications.

Some examples of application of GL in food processing have been reported (Bojsen et al. 2005, Tsukazaki et al. 2012, Moayedallaie et al. 2010). In particular, Lipopan™, which has inherent activity toward phospholipids and glycolipids, has gained interest in the bread-making industry (Gerits et al. 2014). This lipase, derived from the fungus *Fusarium oxysporum*, increases expansion of the gluten network, improving the volume and crumb structure of breads. *Chlorella* is well known as a useful microalga containing proteins, carbohydrates, vitamins, and minerals, and it has long been used as a health additive for human consumption, indicating its safety. Thus, as an alternative GL, ckGL could be also employed in texture improvement of some food materials.

## Electronic supplementary material

ESM 1 (PDF 816 kb)
