# Supplementary material for: A novel galactolipase from a green microalga Chlorella kessleri: purification, characterization, molecular cloning, and heterologous expression
Source: Appl Microbiol Biotechnol. 2018 Jan 3;102(4):1711–23. doi: 10.1007/s00253-017-8713-7 (PMC5794828; doi:10.1007/s00253-017-8713-7)
Supplement: Supplementary file 1 — (PDF 816 kb) [file 253_2017_8713_MOESM1_ESM.pdf]

Applied Microbiology and Biotechnology

# A novel galactolipase from a green microalga *Chlorella kessleri*: purification, characterization, molecular cloning and heterologous expression

Shuhei HASHIRO[1], Koyu FUJIUCHI[2], Daisuke SUGIMORI[2]*, and Hisashi YASUEDA[1]*

[1] *Institute for Innovation, Ajinomoto Co., Inc., 1-1 Suzuki-cho, Kawasaki-ku, Kawasaki 210-8681, Japan*
[2] *Department of Symbiotic Systems Science and Technology, Graduate School of Symbiotic Systems Science and Technology, Fukushima University, 1 Kanayagawa, Fukushima 960-1296, Japan*

*To whom correspondence should be addressed:
Hisashi YASUEDA[1], Tel.: +81-44-245-8554; Fax: +81-44-244-9617; E-mail: hisashi_yasueda@ajinomoto.com
Daisuke SUGIMORI[2], Tel.: +81-24-548-8206; Fax: +81-24-548-8206; E-mail: sugimori@sss.fukushima-u.ac.jp

## Supplementary figures

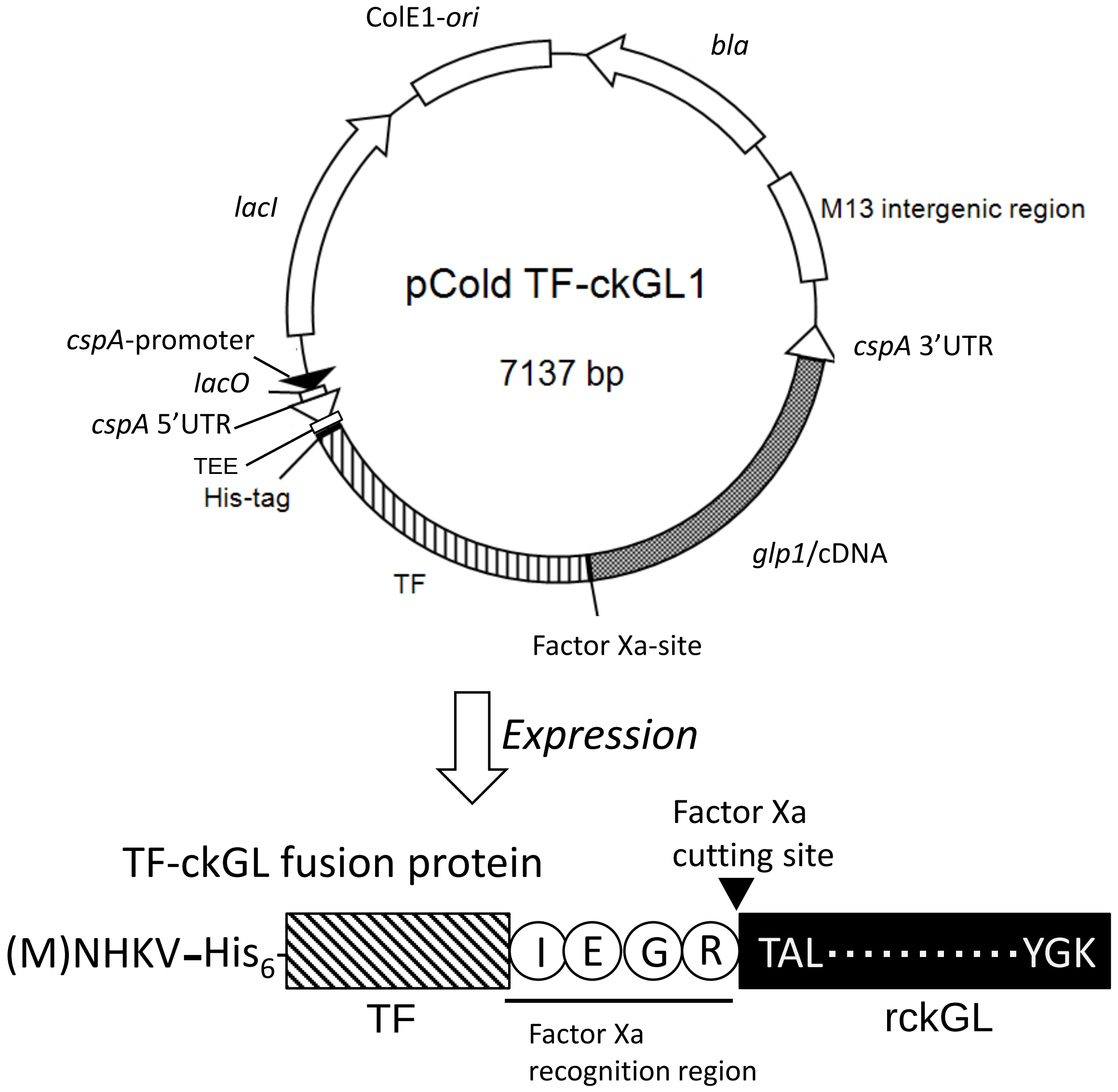


**Figure S1** Structure of the rckGL expression plasmid pCold TF-ckGL1 and the TF-ckGL fusion protein. The fusion protein was produced using the promoter of the *cspA* gene, which encodes a cold shock protein. TEE, translation enhancing element; $His_6$, His-tag; TF, trigger factor; UTR, untranslated region. The recognition region and cutting site of factor Xa are indicated as "I-E-G-R" and a black down-pointing triangle, respectively.

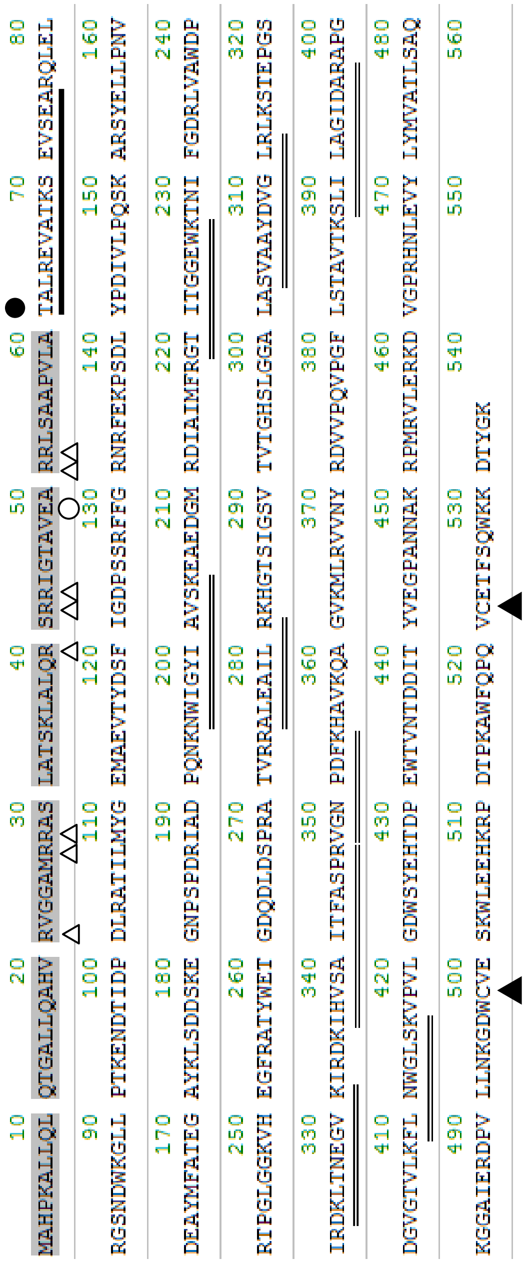


**Figure S2** Amino acid sequence of pre-mature ckGL.

The pre-mature ckGL was composed of 535 amino acids (aa) and a pre-sequence (60 aa) containing a chloroplast transit peptide (cTP, shaded in gray). The initiator Met of pre-mature ckGL was assigned as position +1 of the aa sequence, and the *N*-terminal Thr of mature ckGL at position 61 is indicated with a dot. The N-terminal aa sequence of purified ckGL was detected experimentally (bold underline). Amino acid sequences detected by LC-MS/MS analysis of ckGL were all assigned to corresponding sequences (indicated by double underlines), although the MS-analysis could not distinguish between Leu and Ile residues since they have identical mass. The one negatively charged aa (Glu) and Arg residues in the cTP region are indicated by an open circle and open triangles, respectively. Two Cys residues are indicated by closed triangles.

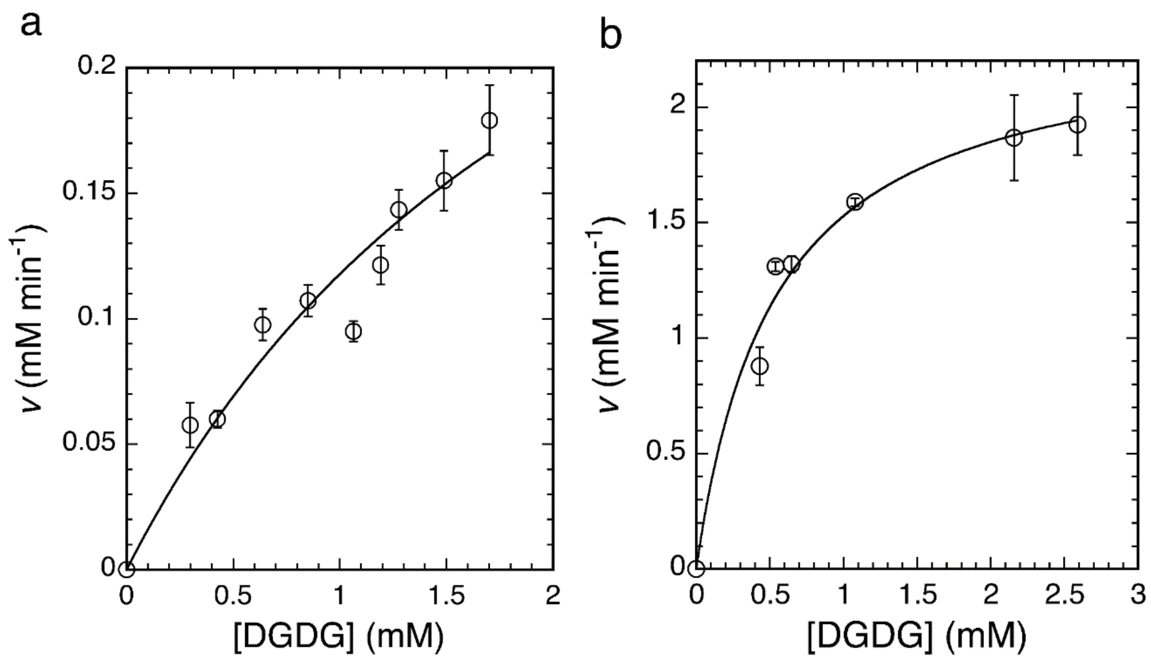


**Figure S3** Steady-state kinetic analyses of (a) the purified native ckGL and (b) rckGL. Each data point represents the mean of three independent determinations with standard errors indicated.

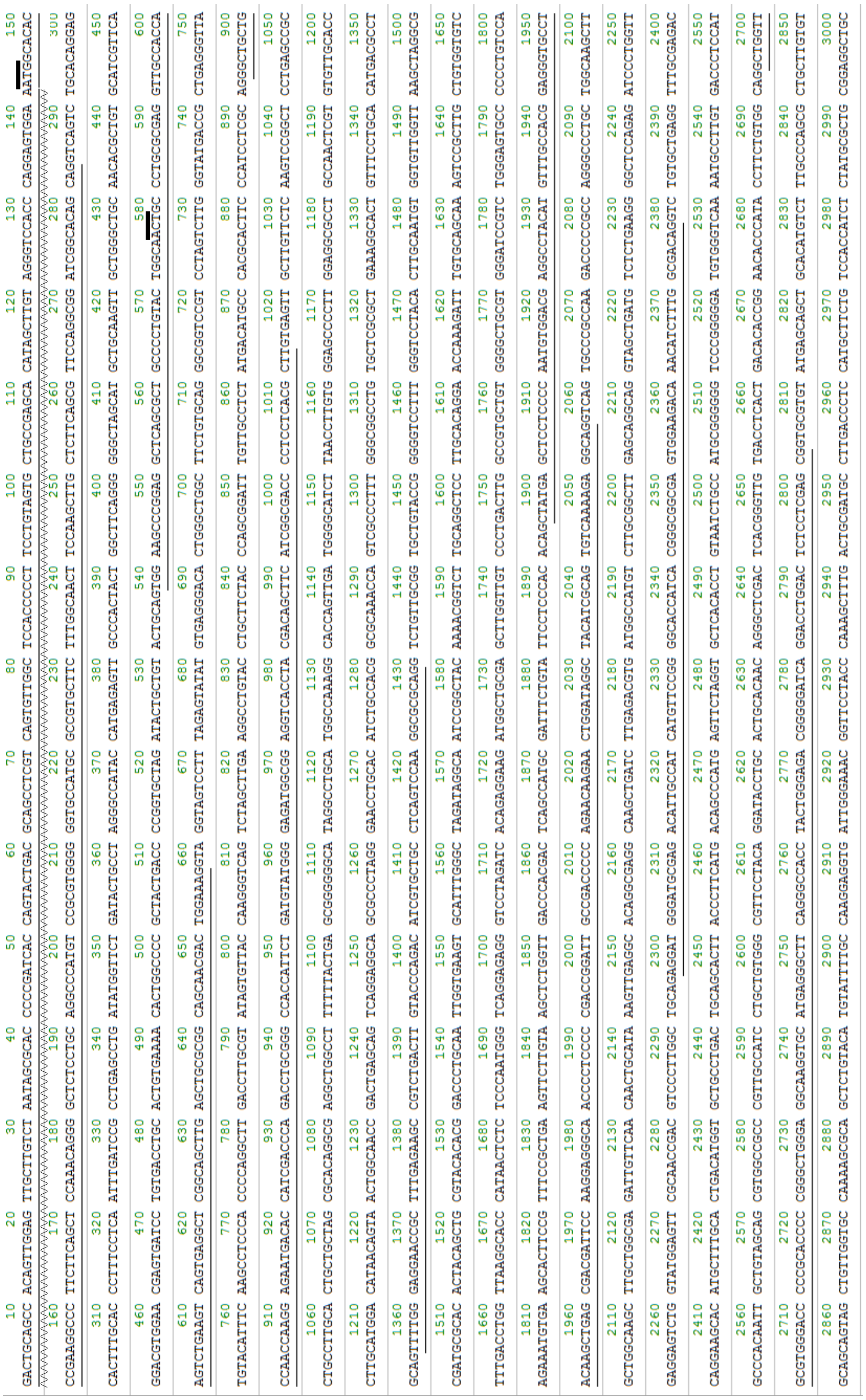

**Figure S4** DNA sequence of *glp1* gene.

The sequence of *glp1* gene contained 10 introns and 11 exons (regions with underlines). The ATG for initiation codon and the ACT codon for the *N*-terminal amino acid (Thr) of mature ckGL are indicated with bold bars. The 5'- and 3'-untranslated regions are underlined with wavy lines.

```
      3010       3020       3030       3040       3050       3060       3070       3080       3090       3100       3110       3120       3130       3140       3150
CGGAACAAGC CTGTACTGCA CTGTATTGTA CGGTACAGTG TGGAGCAGGC CAGCAGGCAA CTACCTTATG GCATGTGCTG CCAGCAGCTG GGGTCCTGCA CCGCTCTACC CCTTTGCTGC TGCACCTCAT CTTGCTGCTT CTCTCAAGAG
      3160       3170       3180       3190       3200       3210       3220       3230       3240       3250       3260       3270       3280       3290       3300
ACGCCAAGCG TTACTCAACG TCCCCACAAT CTTCCTCGGT TCTGACCCTC TGTGGCCTGT TGCCCCTACG ACTGCAGACC GTGCGCCGGG CTCTGGAGGC CATTCTGCGC AAGCATGGCA CTTCGATTGG CTCCGTCACA GTGACAGGCC
      3310       3320       3330       3340       3350       3360       3370       3380       3390       3400       3410       3420       3430       3440       3450
ACAGCCTGGG CGGTGCGCTG GCCAGCGTGG CAGCCTATGA TGTCGGGCTG CGCCTGAAGT CAACCGAACC TGGCAGCATC AGGGACAAAC TGACCAACGA GGGAGTCAAG ATCCGGGACA AGATCCACGT CTCAGCGATA ACCTTTGCGT
      3460       3470       3480       3490       3500       3510       3520       3530       3540       3550       3560       3570       3580       3590       3600
CGCCGCGTGT GGGCAACCCC GACTTCAAGC ATGCTGTGAA GCAGGCGGGG GTGAAGATGC TGCGGGTCGT CAACTACAGG GACGTGGTCC CACAAGTGCC AGGTAGGCGC CCCGGCAGGC TTGCGCTGTG GGGTTGGGTT GTAACTGGCT
      3610       3620       3630       3640       3650       3660       3670       3680       3690       3700       3710       3720       3730       3740       3750
GCAGAGTGGC GCAGGCGGTT GCCTGTGTAG CCGCAGTGTG GCAGCCCCAA TGGGATAGTA CAAATAGGCC TTATGCGTCT AGCAGCCATC CTCATTTCGT AGTCGCACTG ATGCCCTGGG TTGCTTTGAT ATTGCTCCTT TCACAATGCG
      3760       3770       3780       3790       3800       3810       3820       3830       3840       3850       3860       3870       3880       3890       3900
CACCATCACA GAGGTGTGCA GCTTGGCAGC CAGGCATAGC AGGCAACCGG CAAGCAAACC CTAGAACAGC AGACCGCTAA TCAGATGGTG CAGGGTTCCT TAGCAGCGCT CTTTGATTGG TGCAGGATTC CTGAGCACGG CTGTGACCAA
      3910       3920       3930       3940       3950       3960       3970       3980       3990       4000       4010       4020       4030       4040       4050
GAGCCTCATC CTGGCGGGCA TTGATGCCAG GGCTCCAGGT GACGGTGTGG GCACGGTGCT CAAGTTCCTC AACTGGGGAC TCTCCAAGGT TCCGGTGCTC GGTGACTGGT CGTACGAGCA CACCGACCCA GAGGTGCGGA GAGGGTGTGG
      4060       4070       4080       4090       4100       4110       4120       4130       4140       4150       4160       4170       4180       4190       4200
CCCGGTAGGG CTCAAGCTGC TGTGCTCAGC TGCTCAGCAG AGCTGGTCCT GCTTGCTTGC TCCTTCTCTC TCCGGTGGCG CCCGAGGCCA CGTTATAGAC TGCGGTCCAT GATGTGCAAC CGAGGGCGTC TCCGTAACCC ACGCCCCTGG
      4210       4220       4230       4240       4250       4260       4270       4280       4290       4300       4310       4320       4330       4340       4350
CTGGTGTCCT GTGGTTTTCC CAGGGCACCA CTCAAGGCAC GGCCACGGCT GTGTCCTTGA GGTCTGGGCG TATTTATTTA TTTATTTATT TGCTCCTTCT CTTGGCCAGC ACAGCCTGAG TGGTCACAGC AGCATGCTTC TCTTCTACTG
      4360       4370       4380       4390       4400       4410       4420       4430       4440       4450       4460       4470       4480       4490       4500
GCACAGTCCA GAGTGCAGCT GACTTCGCAG CTGCCGGCCA GAGCCTCGGG CCTACAGCCC GGCTACGATC CCGCTCTAAA CCTTGCCTAC TCGCCTGTGC TGCGTGCAGT GGACGGTAAA CACGGACGAT ATCACATATG TGGAGGGGCC
      4510       4520       4530       4540       4550       4560       4570       4580       4590       4600       4610       4620       4630       4640       4650
GGCCAACAAT GCCAAGCGGC CCATGCGCGT GCTGGAGCGC AAGGATGTGG GGCCCCGCCA CAACCTGGAG GTACGACTCC TCACCCACAC TTGGCAAGCC CCTGACTTTG TGGACTACTC TACAGTTCCT GGCCTTCAGT GGGAGGCCTG
      4660       4670       4680       4690       4700       4710       4720       4730       4740       4750       4760       4770       4780       4790       4800
CCCACATGTG TGCTTCTCTG GAGGCCCCTG GACGCCTTAG GCAGACCTTG ACTGTGGCAG CAAGCTTGCT GCCACAGTCA AGGGCAGACT GCGAGCACCT GGAAGGTGTC AGAGGTCCGC CCTCTCGAGA GGCCATGGCA TGGCCCTCCC
      4810       4820       4830       4840       4850       4860       4870       4880       4890       4900       4910       4920       4930       4940       4950
TTTGACCTTC TGCACTCTCC TGTTCGTTGG GCGACTTTCT CAAGGCTACC TGCCTTCAAT CGGCGCAGGT GTACCTGTAT ATGGTGGCGA CGCTGAGTGC CCAGAAGGGT GGGGCCATCG AGCGCGACCC GGTGCTTCTT AACAAGGGCG
      4960       4970       4980       4990       5000       5010       5020       5030       5040       5050       5060       5070       5080       5090       5100
ACTGGTGTGT GGAGTCCAAG TGGCTGGAGG AGCACAAGCG CCCCGACACG CCCAAAGCAT GGTTCCAGCC CCAGGTGTGC GAGACCTTCT CCCAGTGGAA GAAAGATACC TATGGCAAGT GAGGCAGAGC ATGTGAGTGA CGGAGAGATT
      5110       5120       5130       5140       5150       5160       5170       5180       5190       5200       5210       5220       5230       5240       5250
GAACAAAATT ATGGGGTTCA GCAATGGTCG GGCGATGGCG CCTGGACTTC TCCCTGTGGG ATTATTTTAT AGAAGCGATG GACGTGAAGT GCGAGAAGAG AGCATCAGGT ATTGGCTGCT TTGTATGCCA CGCGCCCATG TATCCACACA
      5260       5270       5280       5290       5300       5310       5320       5330       5340       5350       5360       5370       5380       5390       5400
AGTGTATGGC CATGAAGAGG GGGTCTGCAA CCAAGCTTTG GGGGTACAAT ACACAAGTCT TACTGAATTC TGAACATCAC CCCGGAACAT AGACATCCCG CTTATCATTT CCCTTCAGAG TTGAGCTCTG TTTTGCTCCC TTTCAGCTTG
      5410       5420       5430       5440       5450       5460       5470       5480       5490       5500       5510       5520       5530       5540       5550
CTCGGTAACA CTGGCAACAA ATATTTTTCT GATGTTTCTC GACGTGTGTA CATGTCCTCT TGAACTGTTT TCCTGACTCT TGGGCTGCTG ATTAGTGCGT AAGGACGCCA CCTATAACCT GGGGTGGAGC AGGAGTATCC ACAAAATTTT
      5560       5570       5580       5590       5600       5610       5620       5630       5640       5650       5660       5670       5680       5690       5700
GGTCACAGAT AGGTGGTACG ACCTGTGTTA GGATGGGCAA GGTGCAACAT GAAACCTGCC TGGCTGTGGC ATTCTAAAGT CTTGAC
```

**Figure S4** (continued)

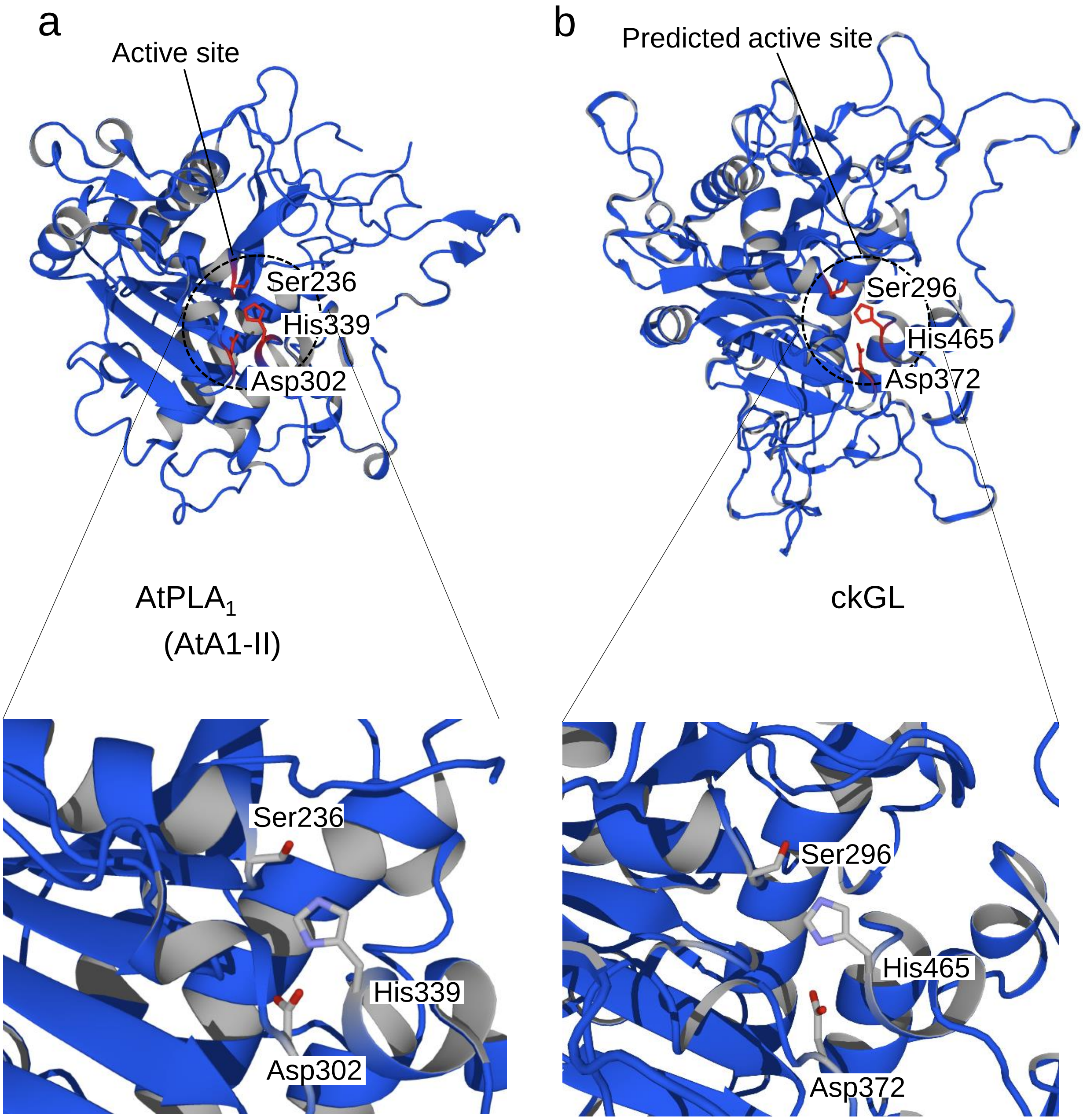


**Figure S5** Crystal and model structures of $AtPLA_1$ and ckGL. (a) $PLA_1$ from *A. thaliana* (PDB ID, 2YIJ) as crystal structure; (b) ckGL as homology-modeled structure. The structures are represented in ribbon models as monomer from the same viewpoint. The putative catalytic triad (Ser, Asp, and His) in the area within black dotted circle is shown in red. In the lower panels, close-up views of the catalytic triads, with oxygen and nitrogen atoms in red and cyan, respectively, are shown as stick models.
